# Factors and Experiences Associated With Unscheduled Hospital Readmission After Lateral Lumbar Interbody Fusion: A Case–Controlled Study

**DOI:** 10.1111/os.70022

**Published:** 2025-03-16

**Authors:** Wangmi Liu, Feng Zhang, Yiqing Tao, Hao Li, Qixin Chen, Fangcai Li

**Affiliations:** ^1^ Department of Orthopedic Surgery The Second Affiliated Hospital, College of Medicine, Zhejiang University Hangzhou China

**Keywords:** adjacent segment disease, lateral lumbar interbody fusion, reoperation, risk factors, unscheduled readmission

## Abstract

**Purpose:**

Understanding the risk factors associated with unscheduled readmission following lateral lumbar interbody fusion (LLIF) is crucial for mitigating the occurrence of these costly events. This study aims to ascertain the incidence and factors of unscheduled hospital readmission subsequent to LLIF.

**Methods:**

A retrospective analysis was conducted on patients who underwent LLIF at our institution from March 2016 to February 2023. Instances of unscheduled hospital readmission after LLIF were meticulously recorded, including baseline demographics, characteristics of spine pathology, surgical interventions, duration between two hospitalizations, and hospitalization costs and duration. Reasons for readmission were categorized based on their etiology. A case–control methodology was employed to compare unscheduled hospital readmission patients against planned readmission patients due to staged surgery. Parametric data were analyzed with a two‐tailed *T*‐test, nonparametric data with the Wilcoxon rank‐sum test, and categorical data with the *χ* ^2^ test.

**Results:**

A total of 1521 patients who received LLIF at our institution were included in the study. A total of 59 patients (3.88%) were unscheduled readmitted due to adjacent segment disease (ASD), cage subsidence, the original surgical segments remaining narrow, spondylodiscitis, and pain. 51 patients (3.35%) experienced reoperation, predominantly attributable to ASD. Compared to planned readmission patients, unscheduled readmission patients tended to be younger, had a lower likelihood of having scoliosis, and were more likely to have short‐segment surgery and higher initial hospitalization costs. Among unscheduled readmission patients, patients receiving short‐segment surgery, as well as those who paid less during the initial hospitalization, demonstrated a higher likelihood of a 90‐day readmission rate.

**Conclusion:**

Our findings indicated the heightened risks of unscheduled hospital readmission after LLIF. Taking targeted measures against these risk factors is expected to reduce the healthcare burden caused by unplanned readmissions in the future.

## Graphical abstract

The research utilized a case–control approach to contrast patients experiencing unscheduled readmissions with those undergoing planned readmissions due to staged surgery. Findings indicated a 3.88% rate of unscheduled readmissions, mainly linked to adjacent segment disease, with 3.35% necessitating reoperation. Factors such as younger age, the absence of scoliosis, opting for short‐segment surgery, and higher initial hospitalization expenses were linked to unscheduled readmissions.

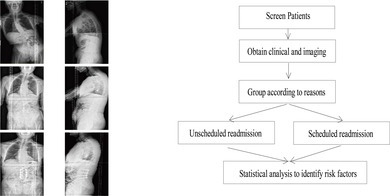

## 1 Introduction

The increasing prevalence of spinal degeneration, affecting older adults disproportionately, is a significant global health challenge due to population aging. Spinal degeneration can lead to conditions like spinal stenosis, spondylolisthesis, and degenerative scoliosis, resulting in chronic pain, decreased mobility, and a diminished quality of life [1]. The rising demand for healthcare services, including surgical interventions, strains healthcare resources and escalates costs [2].

Addressing the challenges posed by population aging and spinal degeneration requires a multifaceted approach that encompasses preventive measures, early intervention strategies, and comprehensive healthcare services tailored to the needs of older adults. Efforts to promote healthy aging, prevent spinal degeneration through lifestyle modifications and targeted interventions, and optimize treatment outcomes are essential to mitigate the adverse effects of spinal degeneration on individuals' quality of life and alleviate the socioeconomic burden on society [3]. Lateral lumbar interbody fusion (LLIF) is an effective surgical technique used in the treatment of various spinal conditions, particularly degenerative disc disease, spondylolisthesis, and spinal deformities. Based on LLIF, we proposed the crenel lateral interbody fusion (CLIF) procedure. This minimally invasive approach involves accessing the spine laterally, usually through a small incision in the patient's side, to remove the damaged disc and insert a fusion cage into the intervertebral space [4]. Studying LLIF is important because it offers effective spinal fusion with potentially shorter recovery times and improved outcomes compared to traditional posterior open surgeries [5]. Clinical practice with LLIF reveals several challenges, primarily related to complications. Neurological complications are prominent, with studies indicating risks of motor and sensory deficits due to the lateral approach traversing near the lumbar plexus. Transient neurologic deficits occurred in 36.07% of cases (95% confidence interval [CI]: 34.74%–37.41%), while persistent neurologic deficits were observed in 3.98% of cases (CI: 3.42%–4.60%) [6].

On the other hand, unscheduled hospital readmission after LLIF may further lead to disability and functional impairment, affecting productivity and participation in the workforce among patients. This, in turn, can lead to decreased economic productivity and increased dependency on social welfare programs. Furthermore, the need for long‐term care and assistance among unscheduled readmission patients places additional strain on families, caregivers, and social support networks [7]. This study aimed to (i) identify the incidence of unscheduled hospital readmission following LLIF procedures, (ii) analyze the risk factors, and (iii) evaluate the causes and treatments related to unscheduled readmissions after LLIF.

## 2 Methods

### 2.1 Study Design and Patient Population

Between March 2016 and February 2023, a retrospective study was conducted at a single institution involving multiple surgeons. The inclusion criteria were (i) patients over 18 years old; (ii) underwent LLIF with or without a posterior procedure during the initial hospitalization for spine diseases; (iii) treated between March 2016 and February 2023 at our institution. Exclusion criteria included a history of prior spinal infection, tumor, or traumatic injury. Unscheduled readmissions were characterized as any non‐elective hospital admissions during the postoperative phase. The number of patients without readmission surgery far exceeded those with unplanned readmissions, making a direct statistical comparison between the two groups not rigorous. Planned admissions for two‐stage surgeries served as the control group. By using planned readmission patients as controls, we aimed to reduce potential biases and confounding factors associated with unplanned readmissions. Approval for the study was obtained from the Institutional Review Board (IRB) of The Second Affiliated Hospital, College of Medicine, Zhejiang University (2024–0305). Due to the retrospective nature of the study, which involves reviewing the medical records of patients who have already undergone treatment, the need for obtaining informed consent was waived by the IRB. The work was reported in line with the STROCSS criteria [8].

### 2.2 Exposure Variables

A thorough review of medical charts was conducted to gather demographic information and clinical profiles of patients. Exposure variables were categorized into preoperative and operative factors. Preoperative variables encompassed demographic characteristics such as age, gender, body mass index (BMI), bone density, presence or absence of scoliosis, modified Frailty Index‐5 (mFI‐5), and smoking and drinking status. As not all patients underwent bone density testing, particularly among younger individuals, Hounsfield unit (HU) values were quantified using data from the Picture Archiving and Communication System, following a methodology outlined in a previous publication [9]. Regions of interest (ROI) were strategically positioned on the midbody axial CT image for the L1 vertebra. Each ROI was delineated as a singular elliptical region, capturing the trabecular bone while carefully avoiding the cortical bone of the vertebral body. The criterion of a mean HU ≤ 110 HU was employed to diagnose osteoporosis [10]. To calculate mFI‐5, the following comorbidities were considered: hypertension requiring medication, chronic obstructive pulmonary disease, congestive heart failure, diabetes mellitus, and totally or partially dependent functional health status [11]. Each item was assigned a single point, and we categorized patients into groups of low frailty (mFI‐5 ≤ 1) and high frailty (mFI‐5 ≥ 2).

Operative variables comprised total instrumented and decompressed levels, length of hospital stay (LOS), and hospitalization costs. Surgery involving three or more segments was considered long‐segment surgery, whereas surgery involving fewer than three segments was categorized as short‐segment surgery.

### 2.3 Outcome Variables

The main focus of interest was the occurrence of unscheduled readmissions after LLIF. Each patient was only counted for their first instance of unscheduled readmission. We recorded days to readmission, cause of readmission, treatment received, readmission stay, and costs. Certain indicators, such as the duration and cost of the second hospitalization, were present in both the unscheduled readmission group and the planned readmission group, which was one of the reasons for comparing these two patient groups.

### 2.4 Surgical Treatment

All patients underwent surgery following the principles of spine surgery, which involved decompression of the nerve and stabilization. For the unscheduled readmission patients, the surgical procedures they underwent during their initial hospitalization were as follows: stand‐alone LLIF, LLIF with lateral plate or rod, and single‐stage or staged LLIF with bilateral pedicle screws. For planned readmission patients, they all underwent stand‐alone LLIF during their initial hospitalization.

### 2.5 Statistical Analysis

Descriptive statistics were utilized to compare patients with unscheduled readmission and those with planned readmission. Continuous variables were summarized using the mean and standard deviation or medians and interquartile ranges, while categorical variables were presented as frequencies. Normality and variance of continuous variables were assessed using the Shapiro–Wilk test and *F*‐test, respectively. Parametric data with equal variance were analyzed using the two‐tailed *T*‐test, while nonparametric data were compared using the Wilcoxon rank‐sum test. Categorical data were analyzed using the *χ* ^2^ test. Kaplan–Meier survival curves and the Log‐rank test were conducted to assess the 90‐day readmission rate among patients with unscheduled readmission. The factors with statistically significant differences from the Log‐rank test were included in the multivariate Cox regression analysis. A significance level of *p* < 0.05 was used. All statistical analyses were performed using R version 4.3.2 (The R Foundation, Vienna, Austria).

## 3 Results

### 3.1 Patient Demographics

Between March 2016 and February 2023, a total of 1521 patients underwent LLIF procedures at our institution. Out of the entire cohort, 59 patients, constituting approximately 3.88% of the total, experienced unscheduled readmissions. During the same period, 34 patients (2.24%) were readmitted for planned surgeries, accounting for scheduled two‐stage procedures following their initial hospitalization. The baseline characteristics of all the patients included in the analysis are listed in Table 1. In continuous variables, only BMI and the second hospitalization costs conformed to normal distribution and homogeneity of variance. These two variables were represented by mean plus standard deviation and analyzed using the *t*‐test. Other continuous variables were represented by the median plus interquartile range and analyzed using the Wilcoxon rank‐sum test. In comparison to patients readmitted for planned surgeries, those experiencing unscheduled readmissions tended to be younger (*p* = 0.032), were more likely to have short‐segment surgery (*p* = 0.009), while being less likely to present with scoliosis (*p* = 0.003). Additionally, they exhibited a longer duration between hospitalizations (*p* < 0.001) and incurred higher initial hospitalization costs (*p* = 0.028). However, the hospitalization costs for unscheduled readmission patients were significantly less than those for planned readmission patients during the second admission (*p* < 0.001). This could be attributed to the fact that planned readmission patients all underwent open surgery, while some unscheduled readmission patients received conservative treatment or minimally invasive procedures.

**TABLE 1:** Demographic and clinical profile of patients.

Variables	Unscheduled readmission (*N* = 59)	Planned readmission *N* = (34)	*t*/*W*/*Χ* ^2^ value	*p*
Gender			2.993^a^	0.084
Female	29 (49.15%)	23 (67.65%)		
Male	30 (50.85%)	11 (32.35%)		
Age	68 (62–72)	71 (65.75–75)	1272^b^	**0.032**
BMI	24.85 ± 3.45	24.10 ± 3.55	−0.982^c^	0.330
Osteoporosis	29 (49.15%)	21 (61.76%)	1.380^a^	0.240
mFI‐5 ≥ 2	24 (40.68%)	14 (41.18%)	0.002^a^	0.963
Scoliosis	18 (30.51%)	21 (61.76%)	8.654^a^	**0.003**
Short‐segment surgery	39 (66.10%)	13 (38.24%)	6.795^a^	0.009
Duration between hospitalizations	111 (56–446)	42.5 (26.25–67.25)	442^b^	**< 0.001**
Initial LOS	13 (8–17)	10 (9–13)	829^b^	0.165
Initial costs	58260.64 (38128.80–96253.63)	54733.75 (44543.56–58934.36)	727^b^	**0.028**
Second LOS	10 (7–15)	11 (8–11)	1039^b^	0.776
Second costs	37771.44 ± 25216.45	57392.58 ± 21012.88	4.025^c^	**< 0.001**

*Note:* Bold font indicates *p*‐values less than 0.05.

Abbreviations: BMI, Body mass indes; LOS, Length of hospital stay; mFI‐5, modified Frailty Index‐5.

^a^ *Χ* ^2^ value.

^b^ *W* value.

^c^ *t* value.

### 3.2 Readmission Causes and Treatments

**FIGURE 1:**
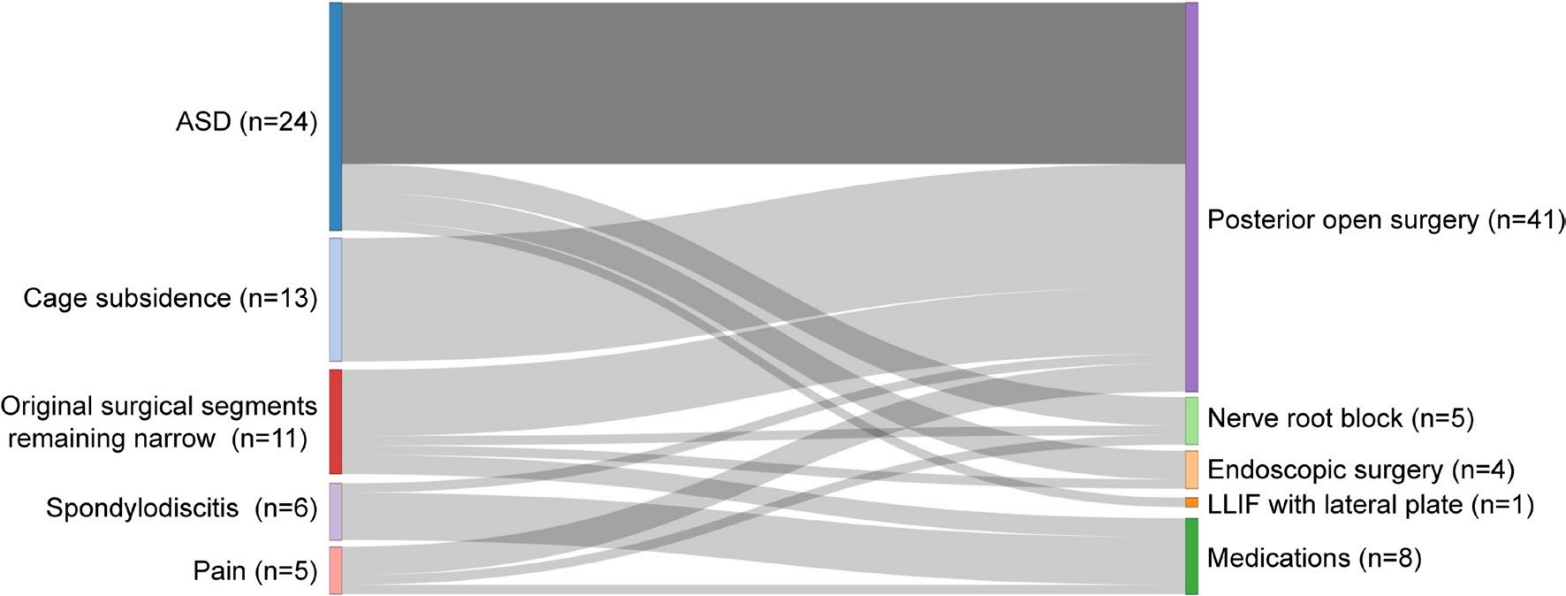
Allocation of treatments to unscheduled readmission patients.

Among the 59 unscheduled readmission patients, the distribution of surgical treatment modalities during the first hospitalization was as follows: 19 patients received stand‐alone LLIF, 7 patients received LLIF with lateral plate or rod, and 33 patients received single‐stage or staged LLIF with bilateral pedicle screws. As shown in Table 2, the most common reason for unscheduled readmissions was adjacent segment disease (ASD), followed by cage subsidence and persistent stenosis at the index surgical segments. During readmission, the most commonly administered treatment was posterior open surgery (Table 3). Additionally, five patients underwent nerve root blockade, four patients received endoscopic minimally invasive treatment, and one patient underwent LLIF with lateral plate fixation. Therefore, a total of 51 unscheduled readmission patients (3.35%) underwent subsequent surgical treatment. The remaining 8 unscheduled readmission patients received medical treatment, including antibiotics and analgesics. All unscheduled readmission patients showed improvement after receiving the appropriate treatment (Figure 1).

**TABLE 2:** Cause of unscheduled readmissions after LLIF.

Cause	Number
ASD	24 (40.68%)
Cage subsidence	13 (22.03%)
Original surgical segments remaining narrow	11 (18.64%)
Spondylodiscitis	6 (10.17%)
Pain	5 (8.48%)

Abbreviations: ASD, Adjacent segment disease; LLIF, lateral lumbar interbody fusion.

**TABLE 3:** Management of unscheduled readmissions after LLIF.

Treatment received	Number
Posterior open surgery	41 (69.49%)
Nerve root block	5 (8.47%)
Endoscopic surgery	4 (6.78%)
LLIF with lateral plate	1 (1.69%)
Medications	8 (13.57%)

Abbreviation: LLIF, lateral lumbar interbody fusion.

### 3.3 Factors Associated With 90‐Day Unplanned Readmission

**FIGURE 2:**
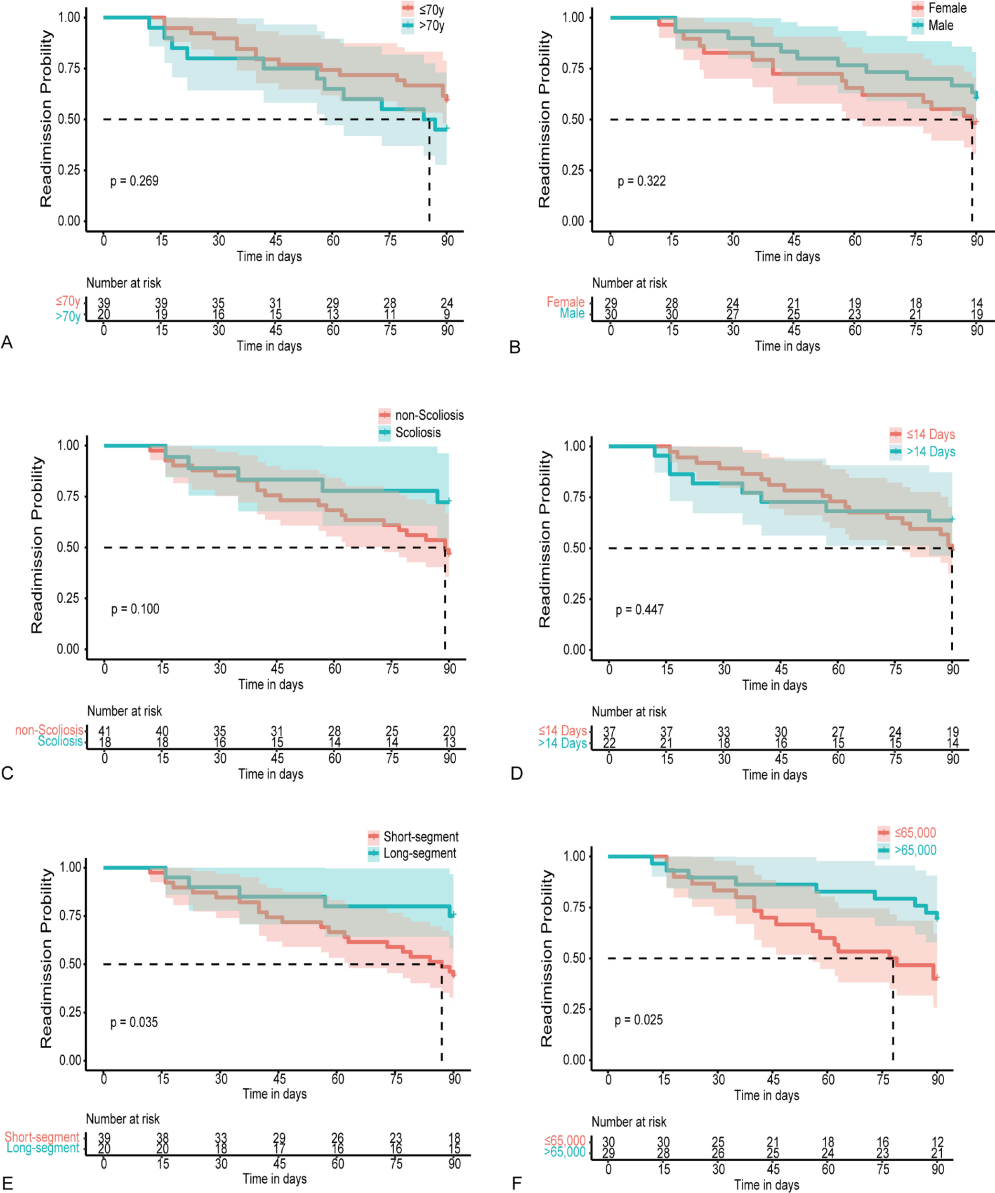
Kaplan–Meier plot of 90‐day readmission among unscheduled readmission patients. (A) Age groups analysis. (B) Gender groups analysis. (C) Scoliosis groups analysis. (D) LOS groups analysis. (E) Segment groups analysis. (F) Cost groups analysis. The dotted line represents median readmission interval.

According to Table 1, the median interval was 111 days for unscheduled readmission. Therefore, we explored the factors influencing unscheduled readmission within 90 days using Kaplan–Meier survival curves, including age, gender, presence of scoliosis, initial LOS, number of operated segments, and initial hospitalization costs. For continuous variables, age was divided into two groups using 70 as the cutoff, operated segments were categorized into two groups using 3 as the cutoff, initial LOS was divided into two groups using 14 as the cutoff, and initial hospitalization costs were categorized into two groups using 65,000 as the cutoff. Compared to patients with long‐segment surgery, those with short‐segment surgery demonstrated a higher 90‐day unscheduled readmission rate (Figure 2E; *p* = 0.035). Additionally, compared to patients with higher initial hospitalization costs, those with lower costs had a higher 90‐day unscheduled readmission rate (Figure 2F; *p* = 0.025). The results of the multivariate Cox regression analysis indicated that these two factors still showed statistical significance, with a *p* value of 0.032 for short‐segment surgery and a *p* value of 0.043 for initial hospitalization costs.

## 4 Discussion

### 4.1 Findings

Our findings highlight the importance of recognizing and addressing risk factors associated with unscheduled readmission following LLIF procedures. Factors such as younger age, non‐preexisting scoliosis, and more initial hospitalization costs were identified as significant predictors of unscheduled readmission. Strategies aimed at optimizing patient selection, perioperative care, and postoperative monitoring may help mitigate the risk of unscheduled readmission and improve overall patient outcomes.

### 4.2 Risk Factors and Countermeasures

According to conventional impression, older patients are often perceived to be at a higher risk of readmission. This perception is grounded in the belief that advancing age correlates with a greater likelihood of experiencing multiple chronic conditions, reduced physiological resilience, and increased susceptibility to adverse events. Consequently, older individuals may face challenges in managing their health conditions and may require more extensive medical interventions, leading to a higher likelihood of hospital readmissions [12, 13]. In the context of lumbar decompression, advancing age has been found to be correlated with an increased likelihood of readmissions within 30 days [14]. However, an alternative study suggests that patients under the age of 65 have a higher probability of requiring reoperation within 30 days following their initial surgery for lumbar degenerative disease and of experiencing readmission within 90 days post‐surgery. This observation underscores the importance of considering age as a potential risk factor for early reoperation and readmission among surgical patients [15]. Similarly, independent risk factors associated with emergency department visits within 90 days after elective spine surgery encompass individuals of a younger age bracket [16]. Therefore, it is important to acknowledge that while age is a significant factor, it is not the sole determinant of readmission risk. Other factors such as comorbidities, socioeconomic status, access to healthcare, and the quality of post‐discharge care also contribute to readmission rates [17]. In our study, there was no significant difference observed in the prevalence of frailty between the planned readmission group and the unscheduled readmission group. The age difference between the two groups may be attributed to the subconscious perception of the surgeons that older patients require more postoperative recovery time. During the postoperative period, these patients were discharged after their first stage surgery, LLIF. Thus, while age serves as an important consideration, a comprehensive assessment of multiple factors is necessary to effectively evaluate and mitigate the risk of unscheduled hospital readmissions in older patients.

According to our previous research, some patients with degenerative scoliosis would experience changes in their Lenke‐Silva classification following LLIF surgery. The change in classification is related to the use of anterior column release and hyperlordotic cage [5]. Therefore, it can lead to a reduction in the number of posterior fusion segments during the second stage, achieving satisfactory improvements in the visual analog scale and Oswestry Disability Index. Another study has similarly demonstrated the staged treatment approach is less invasive, resulting in a decrease in the number of required posterior fixation segments and a reduced need for osteotomy [18]. This strategy not only minimized surgical intervention but also led to improved patient outcomes and reduced postoperative complications [19]. Our institution now commonly adopts a staged surgical approach for the treatment of degenerative scoliosis. For elderly or less tolerant patients, we discharge them to recover for a period of time before readmitting them for the second stage surgery. Therefore, there was a higher proportion of patients with degenerative scoliosis who needed long‐segment surgery in the planned readmission group. Patients readmitted according to schedule only underwent stand‐alone LLIF surgery during their initial hospitalization; hence, their initial hospitalization costs were relatively lower.

Patients who underwent short‐segment surgery exhibited a higher rate of unscheduled readmissions within 90 days compared to those who underwent long‐segment surgery. This suggests that the extent of the surgical procedure may influence the likelihood of unscheduled readmissions shortly after surgery. The dissimilarity between the two groups stemmed from the fact that the short‐segment surgery group demonstrated a higher susceptibility to early instances of cage subsidence. A previous study comprised a total of 365 patients who underwent LLIF and reported that 0.8% encountered cage subsidence [20]. In our study, the probability of cage subsidence was 0.85% (13/1521). In a systematic review, the range of patients experiencing subsidence varies across traditional posterior procedures as follows: for posterior lumbar intervertebral fusions (PLIF), it ranges from 7.4% to 31.8%; and for transforaminal lumbar interbody fusion (TLIF), it ranges from 0.0% to 51.2% [21]. There is a significant correlation between cage size and the external force necessary for a 5 mm subsidence. LLIF constructs exhibit greater stiffness and demand a higher yield force compared to TLIF constructs. Longer bullet cages experience only minor increases in yield force, while lateral cages necessitate notably higher yield force [22]. Similarly, the cage used in LLIF is positioned to encompass the epiphyseal ring, aiming to mitigate cage subsidence [23].

ASD was the primary reason for unscheduled readmissions in this study. However, LLIF is more advantageous in preventing the development of ASD compared to TLIF [24]. In our study cohort, the probability of ASD occurrence was 1.58%. In a previous study, the occurrence rate of ASD in patients undergoing LLIF for degenerative lumbar conditions was found to be 0.88% per year, with a 95% CI ranging from 0.67% to 1.09% [25]. From a mechanical standpoint, LLIF presents advantages that could potentially result in a reduced incidence of ASD. These benefits include minimal tissue disruption and avoidance of facet joint disruption. Theoretically, the preservation of the posterior ligamento‐muscular complex in LLIF procedures may lead to decreased reliance on bony structures for maintaining alignment and support, as opposed to open posterior spine surgeries. One year following the surgical procedure, a notable reduction in muscle density of the multifidus is detected solely among patients who underwent PLIF. Conversely, individuals who underwent LLIF with posterior percutaneous screw placement do not exhibit a significant decrease in multifidus muscle density [26]. This discrepancy in muscle density alterations suggests a potential advantage of LLIF procedures in preserving the integrity and function of the multifidus muscle compared to traditional open PLIF/TLIF surgeries. Moreover, facet joint disruption, particularly at the ends of the fusion construct or in cases of preoperative degenerative facet pathology, has been identified as a potential risk factor for ASD [27]. For the treatment of ASD, we primarily opted for open posterior surgery, with only one case opting for LLIF combined with a lateral plate. According to a biomechanical investigation, LLIF significantly decreases the range of motion (ROM) in all planes. The addition of a plate or rod to LLIF enhances stability compared to LLIF performed alone. The inclusion of posterior instrumentation results in the greatest increase in stability across all ROM planes [28]. Clinical studies have confirmed that LLIF and TLIF are both viable approaches for restoring radiographic alignment and achieving successful clinical outcomes in patients with ASD after prior lumbar fusion surgery. LLIF further offers the potential for reduced perioperative morbidity and shorter LOS while maintaining safety and efficacy [29].

### 4.3 Strengths and Limitations

The study's strengths include its thorough data collection from medical charts, ensuring a detailed analysis of potential risk factors. By using planned admissions for two‐stage surgeries as a specific control group, the researchers reduced potential biases. The study also employed a rigorous statistical approach, including multiple tests and analyses. Furthermore, the research addresses the significant clinical issue of unscheduled readmissions following LLIF and identifies modifiable risk factors. By contributing valuable insights and focusing on targeted interventions, this study enhances the quality of care for patients undergoing LLIF surgery.

On the other hand, this study has several limitations that warrant consideration. Firstly, the data was obtained from a single institution, which may limit the generalizability of the findings. Conducting a larger multicenter study with a larger sample size would be beneficial to validate the results more comprehensively. Secondly, the determination of unplanned readmissions relied on the review of electronic medical records, which might have led to an underestimation of the overall readmission rate by not capturing patients readmitted to other healthcare facilities. Thirdly, the lack of statistically significant differences in certain indicators may be attributed to insufficient statistical power, likely due to the relatively small sample size of the readmitted patient cohort. Therefore, caution should be exercised when interpreting the results, and future research should aim to address these limitations to provide more robust evidence.

## 5 Conclusion

Postoperative unscheduled readmission following LLIF procedures poses challenges for both patients and healthcare providers. By identifying and addressing modifiable risk factors, efforts can be made to reduce the incidence of readmission and enhance the quality of care provided to patients undergoing LLIF surgery. Further research is warranted to validate these findings and develop targeted interventions to minimize postoperative readmissions in this patient population.

## Author Contributions

Writing – original draft: Wangmi Liu and Feng Zhang. Supervision: Fangcai Li. Formal analysis: Wangmi Liu and Yiqing Tao. Data curation: Hao Li. Writing – review and editing: Qixin Chen and Fangcai Li. The authors read and approved the final manuscript.

## Conflicts of Interest

The authors declare no conflicts of interest.
